# Repeated spontaneous remission of acute myeloid leukemia in response to various infections: a case report

**DOI:** 10.1186/s12879-023-08108-z

**Published:** 2023-04-06

**Authors:** Osamu Imataki, Tomoya Ishida, Jun-ichiro Kida, Makiko Uemura, Haruyuki Fujita, Norimitsu Kadowaki

**Affiliations:** Division of Hematology, Department of Internal Medicine, Faculty of Medicine, Kagawa University, 1750-1 Ikenobe, Miki-town, Kita-county, Kagawa 761-0793 Japan

**Keywords:** Spontaneous remission, Acute myeloid leukemia, Infection, Case report

## Abstract

**Background:**

Acute myeloid leukemia (AML) is a progressive hematological malignancy that can be fatal when left untreated. However, spontaneous remission is rarely observed in the presence of infectious diseases.

**Case presentation:**

We treated an 80-year-old woman with AML who spontaneously underwent remission after infections. Spontaneous remission was observed after each of three independent clinical infections caused by different pathogens—nontuberculous *Mycobacterium* infection, pulmonary aspergillosis, and *Escherichia coli* bacteremia. All infections were treated promptly with antimicrobials. *Mycobacterium avium* infection was treated with azithromycin, rifampin, and ethambutol. Pulmonary aspergillosis was treated with itraconazole followed by voriconazole. *E. coli* infection was treated with meropenem. During each infectious episode, leukemic cells disappeared from the patient’s peripheral blood and pancytopenia improved without routine blood transfusion. These clinical effects lasted for several months. The patient has survived for > 2 years beyond the median survival time of end-stage AML. Thus, this case represents an immunological antileukemic effect of systemic infections.

**Conclusions:**

We have discussed a common mechanism of spontaneous remission of AML without chemotherapy, clinically exhibited by infection immunology. We believe that infections exert a limited immunological effect against AML, which may prolong survival among elderly individuals with AML.

## Background

Acute myeloid leukemia (AML) is a fatal and progressive hematological malignancy [1]. Spontaneous regression (SR) is observed as a rare phenomenon in AML, and some contributing factors for AML SR have been reported [2, 3]. Infectious diseases are an important and common cause of SR [4, 5]. The causative mechanisms of SR in AML are immunological in nature [4, 5], including cellular immunity, serological inducers, cytokine pathways, and indirect immunological mechanisms. We experienced a case of repeated spontaneous remission of AML associated with systemic infections.

## Case presentation

An 80-year-old Japanese woman was diagnosed with AML with myelodysplasia-related changes (AML/MRC) via bone marrow examination. She was referred to our hospital with the complication of gradually developing bicytopenia (leukocytopenia and thrombocytopenia), as determined via peripheral blood examination. She had a history of bipolar disorder, which was diagnosed at the age of 78 years, and was treated in the outpatient department of the psychiatric clinic at our hospital. She had no medical history of chemotherapy or radiotherapy. Moreover, she had no history of precedent immunological diseases, such as collagen diseases. She did not receive any immunosuppressive therapy. She had no previous medical history of allergies. However, she had a history of smoking from the age of 20 to 50 years.

**Fig. 1:**
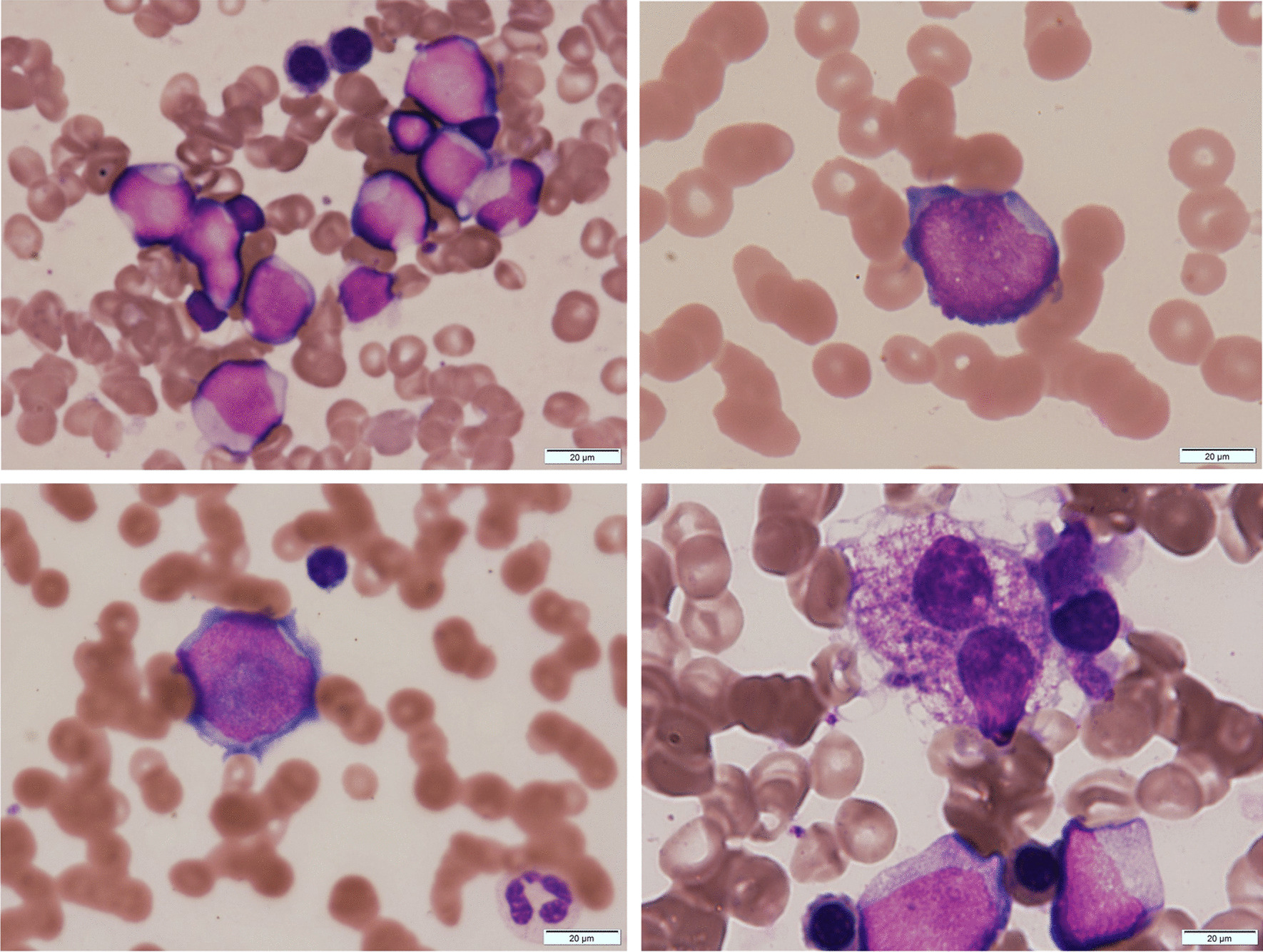
Bone marrow findings of the case. The leukemic cell morphology includes basophilic cytoplasm with deformed nuclei (upper panels). Erythrocytes, granulocytes, and megakaryocytes, all showed dysplastic changes. Erythrocytes showed megaloblastic changes, and granulocytes were hypogranular and agranular (bottom left panel). Micromegakaryocytes were also observed (bottom right panel). All images were acquired using May Grunwald–Giemsa stain at ×100 magnification

Laboratory findings at the first visit of the patient to our hospital were as follows: mild bicytopenia, decrease in white blood cell count to 1600/μL (neutrophils, 31.0%; eosinophils, 2.5%; basophils, 0.0%; lymphocytes, 62.5%; monocytes, 3.0%; and blasts, 0.5%), hemoglobin level of 12.3 g/dL, and decrease in platelet count to 67,000/μL. Mild macrocytic change (mean corpuscular volume, 101.3 fL) was also noted. Biochemical findings, including CRP (0.09 mg/dL) and LDH (192 U/L) levels, showed no abnormalities. The level of *WT1* mRNA, a biomarker, increased to 22,000 (normal range < 50) copies/mL. Bone marrow cell count clonally increased to 4.01 × 10^4^/μL; megakaryocyte count decreased to 18/μL, and blast count increased to 45.4%. She exhibited morphological abnormalities with dysplastic changes, including pseudo Pelger–Huet anomaly, degranular neutrophils, micromegakaryocytes, and ringed sideroblasts, in the three lineages of granulocytes, megakaryocytes, and erythroblasts (Fig. 1). Results of screening for chimeras—major BCR-ABL1, minor BCR-ABL1, PML-RARA, RUNX1-RUNX1T1, CBFB-MYH11, DEK-NUP214, NUP98-HOXA9, ETV6-RUNX1, TCF3-PBX1, STIL-TAL1, KMT2A-AFF1, KMT2A-AFDN, KMT2A-MLLT3, and KMT2A-MLLT1—were negative. The chromosomal test revealed a defective del(5q) clone [6/20]. She was diagnosed with AML/MRC, according to the WHO classification, and M2, according to the FAB classification. According to a consensus of geriatric medicine, owing to her age and underlying illness, she was treated with supportive therapy, such as blood transfusion therapy, instead of aggressive therapy.

**Fig. 2:**
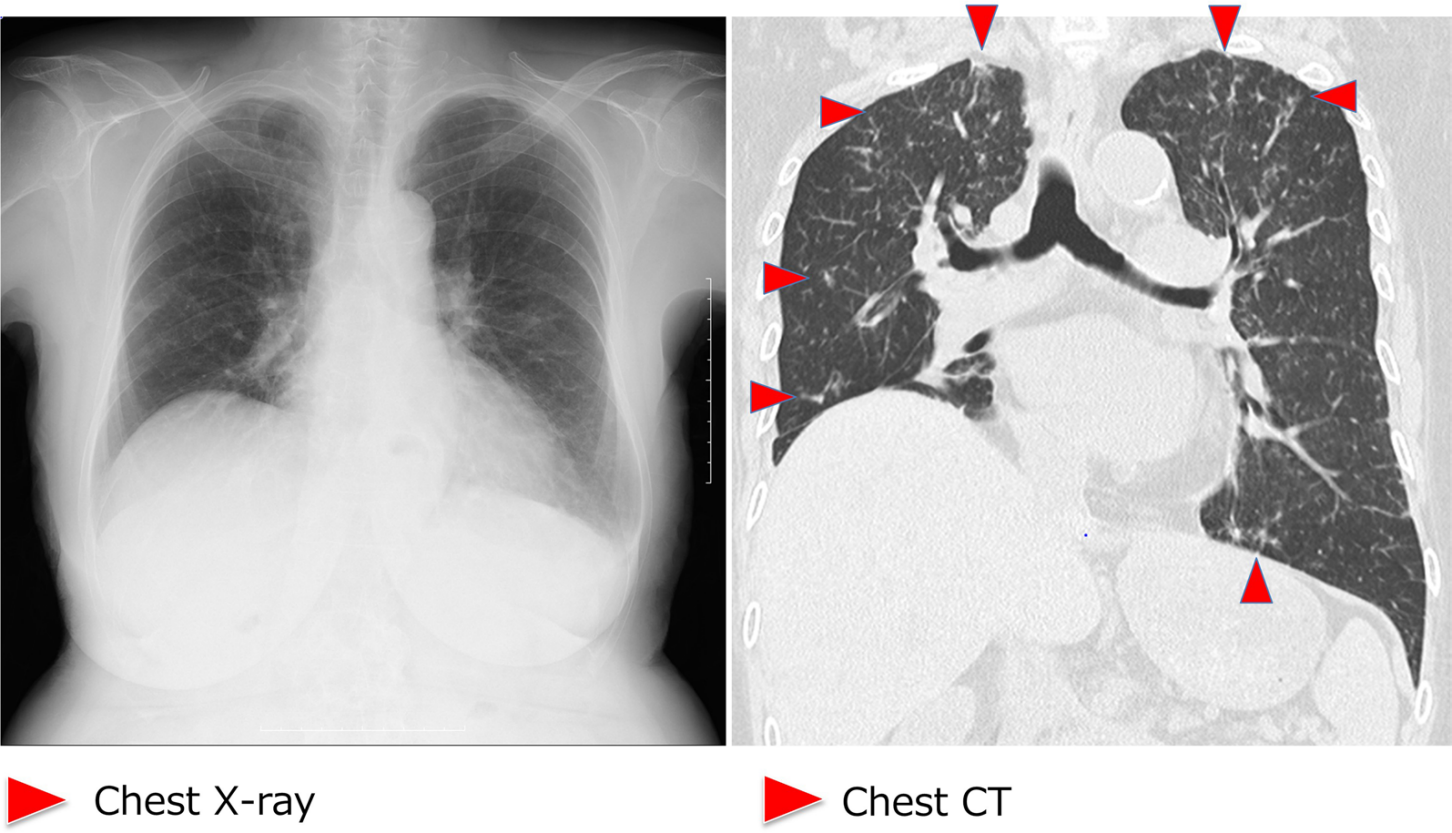
Chest X-ray and chest CT imaging of the non-tuberculous *Mycobacterium*-infected lungs. Images show multiple granular infiltration in the pleural side of all lung fields. Red arrow-heads indicate infiltration

**Fig. 3:**
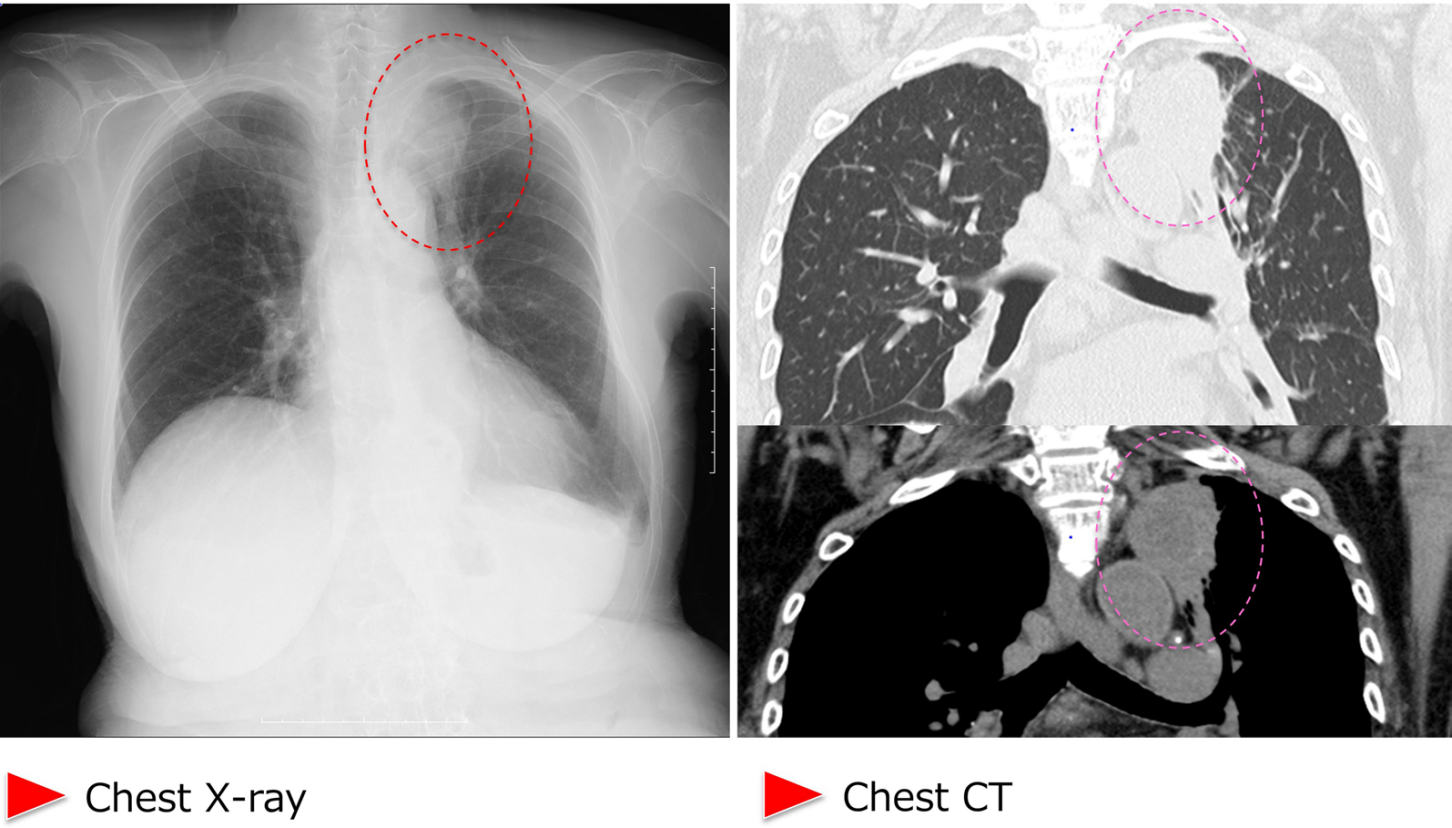
Chest X-ray and chest CT imaging of lungs with pulmonary aspergillosis. Images show a 43 mm nodular shadow in the upper lobe of the left lung. The dotted circle indicates the nodular shadow

**Fig. 4:**
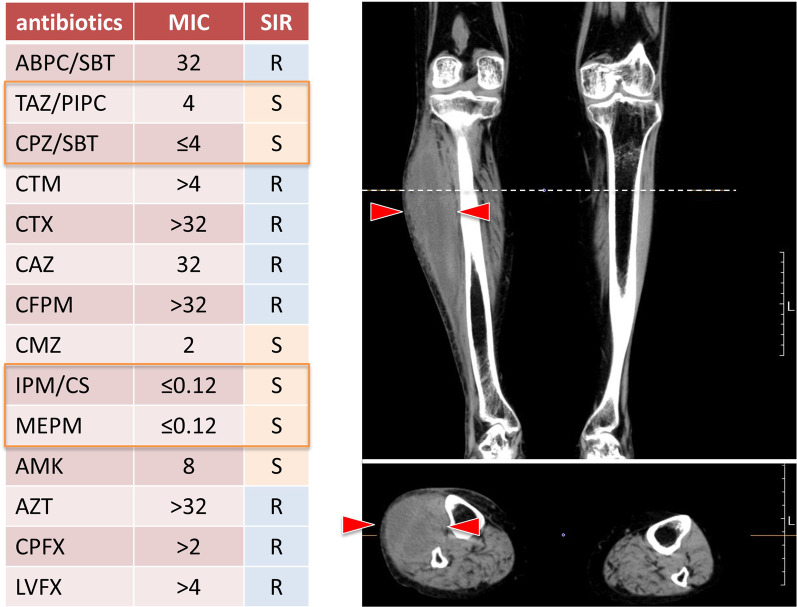
CT imaging of the right lower leg of the patient with *Escherichia coli* cellulitis and subcutaneous abscess with drug susceptibility. The CT scan of the patient’s lower leg revealed an abscess in the lateral side (indicated by red arrow-heads). *E. coli* was detected as the causative pathogen through the culture of blood and subcutaneous abscess. The table on the left shows the antimicrobial susceptibility of the isolated *E. coli*

During supportive blood transfusion therapy, her condition was complicated by the following three infectious diseases: nontuberculous *Mycobacterium* (NTM) infection, pulmonary aspergillosis, and *Escherichia coli* bacteremia. The course of each of these diseases is summarized as follows. (1) NTM: In August 2020, she complained of hypoxemia (SpO_2_ level, 91%) and fever (37.5–37.9 °C). She visited the outpatient clinic at our hospital, and initial chest X-ray revealed multiple small granular shadows in both lungs (Fig. 2). She was positive for anti-MAC antibodies, and PCR was positive for *Mycobacterium avium* in the sputum. We thus diagnosed her with NTM infection. She received triple anti-*Mycobacterium* (azithromycin, rifampin, and ethambutol) treatment for 2 weeks. Consequently, the pulmonary granular shadows reduced, hematopoiesis recovered (with normalized platelet and erythrocyte counts), and precursor cell count in the peripheral blood decreased. This spontaneous remission status persisted for 3 months. Bone marrow examination was performed during the first spontaneous remission. Blast count was 4.6%, and chromosomal abnormality with del(5q) were detected in 2 of 20 analyzed cells (10%). Background myelodysplasia persisted, with hypogranular neutrophils, pseudo Pelger–Huet anomaly, megaloblastoid changes, ringed sideroblasts, and micromegakaryocytes. Flowcytometry detected a scanty of stem cell clones (1.8%) expressing abnormal phenotypes; HLA-DR^+^, CD13^+^, CD34^+^, CD117^+^, all of these are originally expressed aberrant phenotype. This result indicated that she achieved hematological remission with cytogenetically minor residual disease. (2) Pulmonary aspergillosis: In March 2021, she presented with fever (38.4 °C) and hypoxemia (SpO_2_ level, 93%) again. She was treated with levofloxacin for a few weeks, but it did not relieve her fever. In April 2021, chest X-ray revealed pneumonia, with a 43 mm-diameter nodule in her left upper lobe (Fig. 3). She showed elevated levels of β-d-glucan and *Aspergillus* antigen, suggesting pulmonary aspergillosis. She was treated with itraconazole followed by voriconazole for 20 days, after which inflammation and the lung nodule diminished. Following treatment, hematopoiesis recovered and blasts disappeared from the peripheral blood. The del(5q) clones in the bone marrow were detected in 2 of the 20 analyzed cells. She remained in the state of spontaneous remission for 5 months. (3) *E. coli* bacteremia: In December 2021, she presented to the outpatient clinic with a fever of 38.4 °C; she was positive for blood procalcitonin (1.55 ng/mL). We assumed that she had sepsis and thus hospitalized her on the same day. Upon hospitalization, ESBL-positive *E. coli* was detected in two sets of blood cultures, and meropenem treatment was provided. On day 10 of hospitalization, cellulitis of the right lower extremity developed with neutrophil recovery, and ESBL-positive *E. coli* was detected in the subcutaneous abscess at the same site (Fig. 4). The antimicrobial susceptibility to the isolated *E. coli* is shown in Fig. 4.

Her condition improved with puncture drainage and continued antibiotic treatment. During the course of treatment for bacteremia, hematopoiesis recovered and peripheral blood precursor cell count decreased. The del(5q) clones in the bone marrow were only detected in 1 of the 20 analyzed cells. The frequency of blood transfusions decreased; thus, she was temporarily discharged from the hospital. This partial remission status persisted for 3 months. She is still alive and has survived significantly longer than the median survival time of end-stage AML (2 months) [6].

In summary, regarding the mechanisms of the three infectious diseases, we observed multiple therapeutic effects of infectious diseases on AML:

1. Hematopoiesis recovery and disappearance of peripheral blood precursor cells.
2. Quantifiable improvement in dysplasia.
3. Reduced detection of a specific abnormal clone del(5q), as determined using the chromosomal test and FISH.

**Fig. 5:**
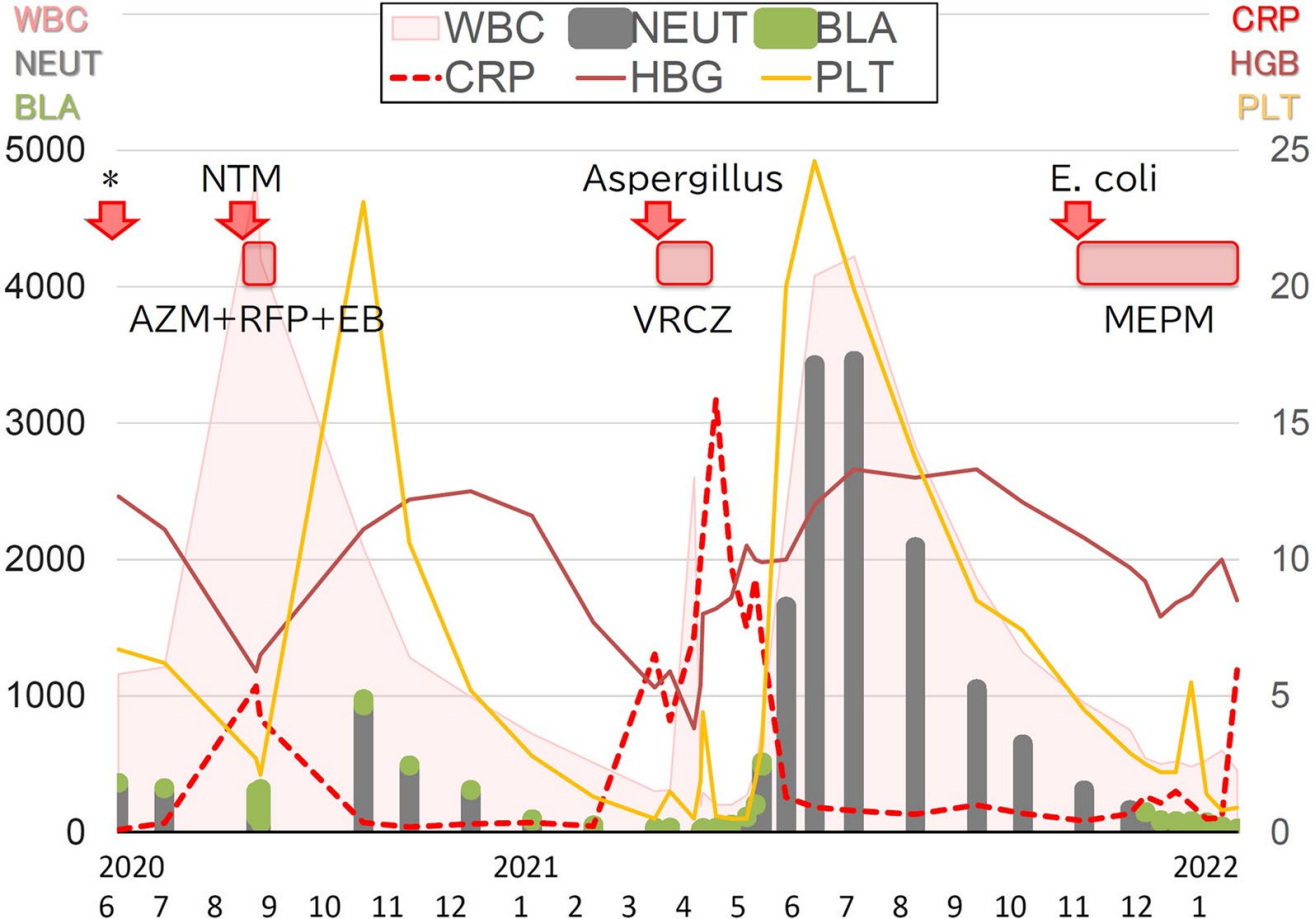
Clinical course of the three infectious diseases causing spontaneous remission of acute myeloid leukemia in our patient. The bar graph shows the white blood cell (WBC) count, neutrophil (NEUT) count, and blast cell (BLA) count on the left axis. The line graph shows the CRP level, hemoglobin (HGB) level, and platelet (PLT) count on the right axis. Recovery of the WBC count, NEUT count, HGB level, and PLT count and a decrease or disappearance of the peripheral blood BLA count were observed after each of the three infections, which persisted for several months. (* indicates the time point of disease diagnosis by bone marrow examination) (*AZM* azithromycin, *EB* ethambutol, *MEPM* meropenem, *RFP* rifampicin, *VRCZ* voriconazole)

Thus, she continued to survive 2 years after AML onset due to clinical infectious diseases caused by various pathogens. Figure 5 presents the course of all spontaneous remissions in this case.

## Discussion and conclusions

We observed three spontaneous remissions associated with clinical infectious diseases in an 80-year-old woman with AML. In each infectious episode, AML ameliorated without any intervention with cytotoxic or molecularly targeted agents. The depth of remission was determined by the decrease in the detection of specific del(5q) clone in cells from 6/20 cells at the onset to 1/20 cells after three infectious episodes. Overall, the spontaneous remissions led to a longer progression-free survival than the median survival time of end-stage AML (2 months). In each episode, the remission status was maintained for 3–5 months, resulting in a total progression-free survival of > 2 years with the help of effective supportive care [7]. The case progression clinically proved infection-related spontaneous remission of AML in response to infection by three different pathogen types: *Mycobacterium avium* (mycobacterium), *Aspergillus* (fungus), and *E. coli* (bacteria).

There have been multiple reports of various infectious diseases ameliorating AML [8, 9]. In our case, systemic infections caused by three different pathogens exerted an antileukemic effect. These antitumor effects may be caused by cell-mediated immunity, cytokines, and growth factors [10–14]. The cytotoxic effects of NK cells [11] and cytotoxic T lymphocytes, which are involved in innate and acquired immunity respectively, are the main contributors to the remission of AML in response to infection [10]. The second most common contributors to the antileukemic effect of infectious diseases are inflammatory cytokines, such as IL-1, IL-6, IL-2, and TNF-α [12, 13]. Some reports have also revealed that G-CSF and GM-CSF exert an antileukemic effect under specific conditions [14].

Although natural remission of AML can be confirmed through genetic or molecular biological analyses, it is a temporary phenomenon [5, 9, 15]. This is because patients with spontaneous remission of AML experience relapse repeatedly without absolute disease resolution. Some genomic analyses have revealed the presence of residual AML clones during spontaneous remission [16, 17]. A clonal study showed that leukemic cells continue to undergo clonal evolution during spontaneous remission [16]. Residual clones may cause AML relapse; however, little is known about the acquisition of resistance by these clones after spontaneous remission. In our case, the molecular maker del(5q) was used for assessing residual diseases. In general, such molecular makers are useful for estimating the depth of remission. No studies have reported an association between spontaneous remission and specific chromosomal abnormality or genetic mutation. However, the deletion (5q) karyotype in MDS is considered a good risk feature. Immunotherapy such as lenalidomide treatment [18] and hematopoietic stem cell transplantation [19] may provide a better response.

With this scientific background, infectious immunity may exert a better effect in MDS. Finally, a sequential antileukemic effect was repeatedly observed over a brief period (20 months). This suggests that an innate or cytokine effect is durable under certain conditions. Interestingly, a previous report proposed that the preleukemic state is observed in older normal individuals and preleukemic remission can occur shortly after chemotherapy [17]. This model suggests the reversion of AML to a preleukemic state during the natural remission period and its conversion to complete leukemia after the diminishing of the immunologic antileukemic effect. According to this model, chronic infection can ameliorate AML temporarily but not permanently.

In conclusion, the patient in our case showed repeated spontaneous remission of AML due to various infectious diseases. The immunological effects of the infectious diseases causing these remissions were clinically confirmed by the decrease in specific chromosomally aberrant clones.

## Data Availability

All data generated or analyzed during this study are included in this published article.

## Abbreviations

AML: Acute myeloid leukemia
MRC: Myelodysplasia-related changes
NTM: Non-tuberculous *Mycobacterium*
SR: Spontaneous regression
